# Longitudinal Flow Cytometric Profiling of CAR-T and Endogenous T Cells Following CD19 CAR-T Therapy

**DOI:** 10.3390/cancers18203374

**Published:** 2026-10-19

**Authors:** Sungyu Jee, Taewon Kang, Hyunjoo Bae, Takho Kang, Jin Jung, Yonggoo Kim, Myungshin Kim, Jae Won Yoo, Gi-June Min, Dong Wook Jekarl, Jong-Mi Lee

**Affiliations:** 1 Department of Laboratory Medicine, Seoul St. Mary’s Hospital, College of Medicine, The Catholic University of Korea, Seoul 06591, Republic of Koreatakho.kang@cmcnu.or.kr (T.K.); bluejin1227@gmail.com (J.J.); yonggoo@catholic.ac.kr (Y.K.); microkim@catholic.ac.kr (M.K.); bonokarl@catholic.ac.kr (D.W.J.); 2 Department of Medical and Biological Sciences, Songeui Campus, The Catholic University of Korea, Seoul 06591, Republic of Korea; 3 Department of Pediatrics, Seoul St. Mary’s Hospital, College of Medicine, The Catholic University of Korea, Seoul 06591, Republic of Korea; hoiring0209@cmcnu.or.kr; 4 Department of Hematology, Catholic Hematology Hospital and Leukemia Research Institute, Seoul St. Mary’s Hospital, College of Medicine, The Catholic University of Korea, Seoul 06591, Republic of Korea; beichest@catholic.ac.kr

**Keywords:** flow cytometry, T-cell differentiation, Tisagenlecleucel, diffuse large B-cell lymphoma, B-cell acute lymphoblastic leukemia, immune effector cell-associated neurotoxicity syndrome

## Abstract

**Background/Objectives:** Longitudinal monitoring after CD19-directed chimeric antigen receptor (CAR) T-cell therapy primarily focuses on CAR-T-cell expansion. However, comprehensive immunophenotypic characterization of both CAR-T and endogenous T cells remains limited. We investigated longitudinal immune dynamics following tisagenlecleucel treatment using multiparameter flow cytometry. **Methods:** Nineteen patients with relapsed or refractory B-cell malignancies (11 diffuse large B-cell lymphoma and eight B-cell acute lymphoblastic leukemia) receiving tisagenlecleucel were prospectively enrolled. Serial peripheral blood samples collected during the first 28 days after infusion were analyzed for CAR-T-cell kinetics, differentiation status, activation and exhaustion marker expression, longitudinal phenotypic changes, as well as associations with treatment response and immune-related toxicities. **Results:** CAR-T-cell expansion predominantly peaked on day 7 after infusion. However, in several patients with B-cell acute lymphoblastic leukemia, delayed peak expansion was observed. At peak expansion, CAR-T-cells were enriched for a central memory phenotype and exhibited significantly higher HLA-DR and PD-1 expression than endogenous T cells. During follow-up, the proportion of central memory T-cells progressively declined, whereas the proportion of terminal effector T-cells increased, which was accompanied by reduced expression of HLA-DR, PD-1, and TIGIT. Despite the distinct activation profiles, CAR-T and endogenous T-cells displayed broadly similar differentiation patterns. Exploratory analyses further demonstrated higher absolute CAR-T-cell counts in patients who developed immune effector cell-associated neurotoxicity syndrome. **Conclusions:** This clinically applicable approach provides immunophenotypic information beyond CAR-T-cell enumeration alone and may facilitate biomarker discovery and longitudinal immune monitoring in patients receiving CAR-T-cell therapy.

## Simple Summary

Chimeric antigen receptor (CAR) T-cell therapy is an effective treatment for several difficult-to-treat B-cell cancers, but the immune changes that occur after treatment are not fully understood. Most monitoring focuses primarily on circulating CAR-T cell counts. In this study, we used multiparameter flow cytometry to monitor both CAR-T cells and endogenous T cells during the first month after tisagenlecleucel infusion. CAR-T cells showed changes in expansion, differentiation, and expression of immune-related markers over time, while their overall differentiation pattern was broadly similar to that of endogenous T cells. Patients who developed neurotoxicity also showed higher absolute CAR-T cell counts in an exploratory analysis. These findings suggest that flow cytometry can provide useful information beyond CAR-T-cell enumeration and may support longitudinal immune monitoring after CAR-T-cell therapy.

## 1. Introduction

CD19-directed chimeric antigen receptor (CAR) T-cell therapy has substantially improved survival outcomes in patients with relapsed or refractory B-cell malignancies, including diffuse large B-cell lymphoma (DLBCL) and B-cell precursor acute lymphoblastic leukemia (B-ALL) [1,2]. Despite these remarkable advances, disease relapse and treatment-related toxicities, particularly cytokine release syndrome (CRS) and immune effector cell-associated neurotoxicity syndrome (ICANS), remain major clinical challenges [3,4]. Numerous clinical and biological factors, including baseline disease burden, inflammatory markers, CAR-T product characteristics, and early CAR-T expansion kinetics, have been proposed to predict treatment efficacy and toxicity [5,6,7]. However, clinically robust and consistently validated biomarkers are still lacking [7,8].

Following CAR-T-cell infusion, in vivo CAR-T expansion is a major factor influencing therapeutic response and treatment-related toxicities [6,9]. Accordingly, longitudinal monitoring of circulating CAR-T cells has become an integral component of clinical management. Several approaches, including molecular assays and flow cytometry, have been adopted to monitor CAR-T-cell expansion in clinical practice [10,11,12]. Notably, a recent survey conducted by the Cellular Therapy and Immunobiology Working Party (CTIWP) of the European Society for Blood and Marrow Transplantation (EBMT) demonstrated the widespread implementation of flow cytometry-based immune monitoring across transplant centers [13].

Beyond CAR-T-cell expansion itself, accumulating evidence suggests that the endogenous immune compartment also contributes to treatment outcomes. Single-cell studies have highlighted that CAR-T therapy is accompanied by dynamic changes in endogenous T-cell populations and that specific T-cell states are associated with CAR-T-cell persistence and treatment response [14,15]. Considering these findings, evaluating both CAR-T cells and endogenous T cells during immune monitoring has become important for understanding the biological determinants of treatment success and toxicity [13,16].

Multiparameter flow cytometry provides a unique advantage of simultaneously quantifying both CAR-T cells and endogenous T cells while characterizing their differentiation, activation, and exhaustion status [10,11,17,18]. This integrated approach provides insights beyond simple CAR-T-cell enumeration by simultaneously assessing engineered and endogenous immune compartments. However, longitudinal clinical studies comprehensively evaluating these immunophenotypic features remain limited.

To address this gap, we performed longitudinal multiparameter flow cytometric immune monitoring in patients receiving tisagenlecleucel. We aimed to comprehensively characterize the dynamic immunophenotypic changes in CAR-T cells and endogenous T cells following CAR-T-cell therapy, thereby providing insights into the immunological mechanisms underlying therapeutic response and treatment-related toxicities.

## 2. Materials and Methods

### 2.1. Patients and Samples

Patients with relapsed or refractory B-ALL (*n* = 8) or DLBCL (*n* = 11) who received tisagenlecleucel (Kymriah^®^; Novartis Pharma AG, Basel, Switzerland) at Seoul St. Mary’s Hospital, Korea, between April 2025 and March 2026 were included. Peripheral blood samples were prospectively collected at predefined time points after CAR-T infusion to evaluate CAR-T-cell kinetics and immune profiling. A total of 54 peripheral blood samples were analyzed, including samples collected on days 3 (*n* = 6), 7 (*n* = 17), 14 (*n* = 13), 21 (*n* = 12), and 28 (*n* = 6) after CAR-T-cell infusion.

### 2.2. Flow Cytometry Analysis

EDTA-anticoagulated peripheral blood samples were processed within 24 h of collection using our routine clinical flow cytometry protocol, which did not include viability staining. For immunophenotyping, peripheral blood aliquots were incubated with the twelve antibodies (Supplementary Table S1) for 15 min at room temperature in the dark. Erythrocytes were subsequently lysed using VersaLyse Lysing Solution (Beckman Coulter, Brea, CA, USA; Ref: A09777). Nucleated cells were washed three times with phosphate-buffered saline (PBS) and centrifuged at 400× *g* for 3 min between washes, resuspended in 150 μL PBS, and immediately acquired on a DxFLEX flow cytometer (Beckman Coulter, Brea, CA, USA). A minimum of 100,000 events were collected per sample. Flow cytometry data were analyzed using Kaluza software version 2.4 (Beckman Coulter, Brea, CA, USA). To support the reliability of CAR-T-cell identification, two CAR-T-positive samples and one CAR-T-negative sample were analyzed using both the study reagent and an alternative CAR detection reagent, demonstrating comparable identification of CAR-positive T cells (Supplementary Figure S1). In addition, seven additional CAR-T-cell-negative samples were evaluated using the study reagent, yielding a total of eight negative controls. Background CAR-positive staining was minimal (median, 0.005%; range, 0–0.31%), and the CAR-positive gating thresholds established using these controls were consistently applied to all study samples (Supplementary Figure S2).

### 2.3. Gating Strategy

Flow cytometric data were analyzed to characterize CAR-T-cell and endogenous T-cell subsets. The hierarchical gating strategy is illustrated in Supplementary Figure S3. Briefly, singlets were identified using forward scatter height (FSC-H) versus forward scatter area (FSC-A) to exclude doublets. Lymphocytes were subsequently gated based on CD45 expression and side scatter (SSC-A) characteristics. Within the lymphocyte gate, CD3+ T cells and CD19+ B cells were identified, and CD3+ T cells were further classified as CAR-positive (CAR-T cells) and CAR-negative (endogenous T cells) based on CAR-FITC staining. The CAR-positive gate was established using CAR-T-negative control samples and applied consistently across samples to minimize nonspecific identification of CAR-positive T cells. Both the CAR-T and endogenous T-cell populations were further subdivided into CD4+ and CD8+ subsets. T-cell differentiation status was assessed using CCR7 and CD45RA expression. Naïve T cells (TN) were defined as CD45RA+CCR7+, central memory T cells (TCM) as CD45RA− CCR7+, effector memory T cells (TEM) as CD45RA− CCR7−, and effector T cells (TE) as CD45RA+ CCR7−. HLA-DR, PD-1, and TIGIT expression were assessed in CAR-T and endogenous T-cell populations using median fluorescence intensity (MFI).

### 2.4. Definition of Peak CAR-T Expansion and Longitudinal Analyses

Peak CAR-T expansion was defined individually for each patient as the time point with the highest absolute circulating CAR-T-cell count among the available longitudinal samples. For longitudinal analyses, 14 patients with at least one post-peak sample were included. The remaining five patients were excluded because their peak expansion occurred at the last available sampling time point (day 7, *n* = 2; day 21, *n* = 2; and day 28, *n* = 1), precluding assessment of post-peak changes. When multiple post-peak samples were available, the latest available follow-up sample was analyzed to characterize immunophenotypic changes during the post-peak phase. Missing scheduled samples were not imputed. Because peak timing and sample availability differed among patients, the exact paired time points varied across patients. Patient-level peak and follow-up time points are provided in Supplementary Table S2.

### 2.5. Statistical Analyses

Statistical analyses were performed with MedCalc software (version 23.5.5; MedCalc Software Ltd., Ostend, Belgium). Unpaired two-sided Mann–Whitney *U* tests were applied to assess the statistical significance between groups of unpaired samples, and the Wilcoxon matched-pairs test was used to assess the statistical significance between groups of paired samples. Differences in categorical variables between the two independent groups were evaluated using Fisher’s exact test. A *p* value < 0.05 was considered statistically significant.

## 3. Results

### 3.1. Patient Characteristics

The baseline characteristics of the 19 enrolled patients are summarized in Table 1. At the time of CAR-T infusion, 15 patients (78.9%) had relapsed disease, and four patients (21.1%) had refractory disease. Patients with B-ALL were substantially younger than those with DLBCL (median age, 13 vs. 59 years, *p* < 0.001). The median interval between leukapheresis and CAR-T infusion was 34 days (range, 26–61), including 35 days (33–46) for DLBCL and 30.5 days (26–61) for B-ALL. Patients with DLBCL had higher WBC, ANC, and LDH levels (*p* = 0.012, *p* = 0.009, and *p* = 0.016 respectively) and lower lymphocyte counts (*p* = 0.032) than those with B-ALL. The absolute number of CD3+ cells collected by leukapheresis was significantly higher in patients with DLBCL than in those with B-ALL (*p* = 0.029). All patients with DLBCL received bridging therapy with polatuzumab vedotin, bendamustine, and rituximab. Among patients with B-ALL, bridging therapy consisted of inotuzumab ozogamicin (*n* = 1), vincristine plus dexamethasone (*n* = 4), vincristine plus dexamethasone and cytarabine (*n* = 2), or vincristine plus prednisolone (*n* = 1). All patients received lymphodepletion with fludarabine and cyclophosphamide before CAR-T-cell infusion. All patients with B-ALL achieved complete remission (CR) at day 30, whereas patients with DLBCL showed heterogeneous responses. Grade ≥ 2 CRS occurred more frequently in patients with DLBCL than in those with B-ALL (63.6% vs. 37.5%), and ICANS was exclusively observed in patients with DLBCL.

### 3.2. Expansion Kinetics of Circulating CAR-T Cells Following Infusion

**Figure 1:**
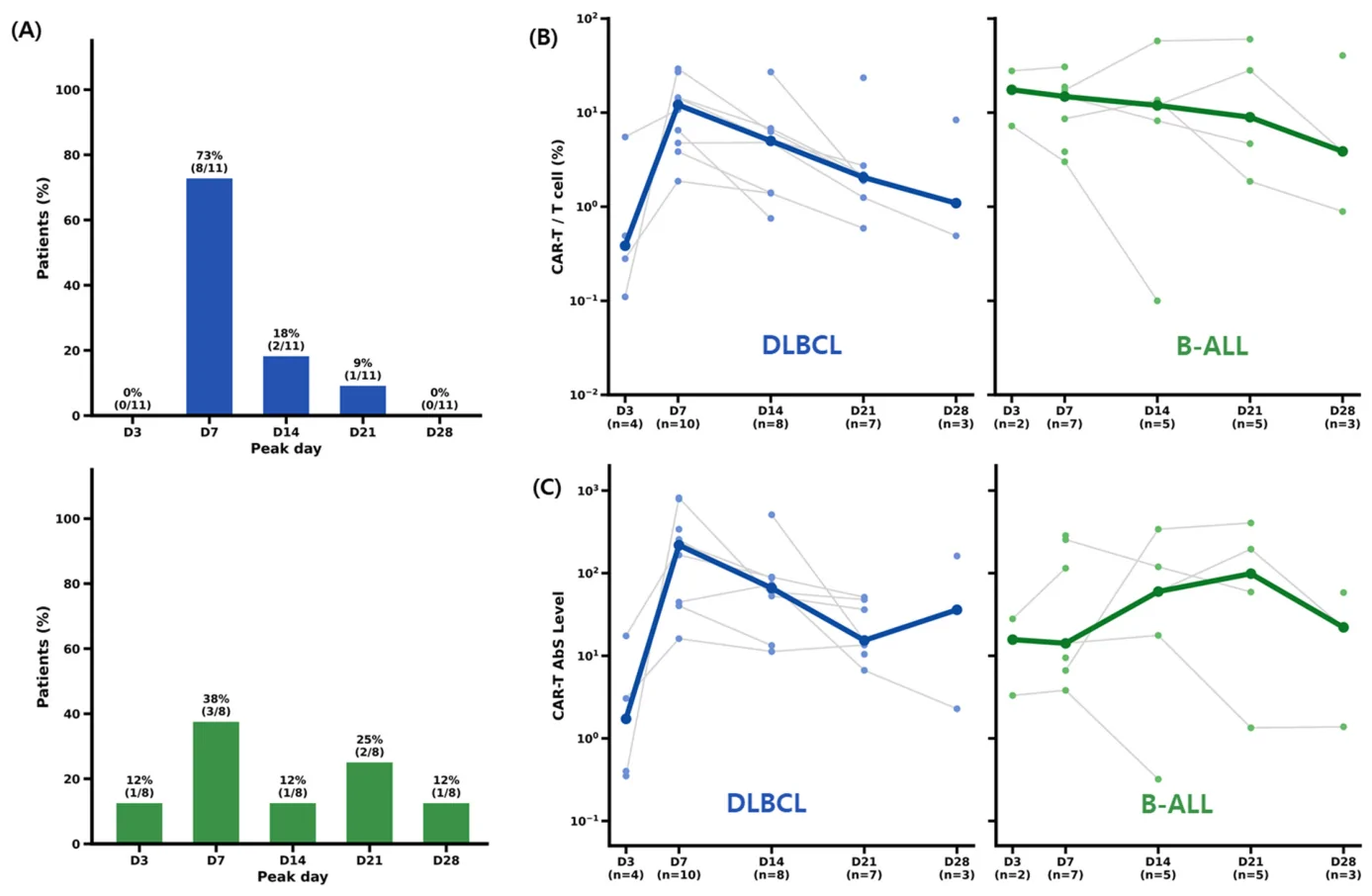
Kinetic profiles and peak expansion day of CAR-T cells by diagnosis groups. (**A**) Distribution of the peak expansion day for DLBCL (*n* = 11) and B-ALL (*n* = 8) patient cohorts. (**B**) Longitudinal kinetics of relative CAR-T-cell percentages among total T cells and (**C**) absolute CAR-T-cell counts (×10^6^/L) during the first 28 days after infusion. Light gray lines represent individual patients, and thick solid lines with circles represent the median values. The number of samples available at each time point is indicated below the *x*-axis.

CAR-T expanded rapidly after infusion. In the overall cohort, the median CAR-T proportion increased from 3.0% (0.33–6.79) on day 3 to 13.46% (4.75–17.28) on day 7 and subsequently declined to 2.48% (0.94–7.24) by day 28. Similarly, the absolute CAR-T counts peaked at day 7 at a median value of 165.96 × 10^6^/L (16.11–256.02). Peak-expansion timing differed between the two disease groups (Figure 1A). Among the patients with DLBCL, 73% (8/11) reached peak expansion on day 7, whereas patients with B-ALL showed greater variability, with peak expansion occurring between days 3 and 28. Delayed peak expansion beyond day 14 was observed exclusively in the B-ALL cohort (Figure 1A–C). Interestingly, the relative CAR-T proportion decreased to near day 3 levels by day 28, whereas the absolute CAR-T counts remained higher than those observed at day 3, indicating distinct longitudinal patterns for relative and absolute CAR-T measurements (Supplementary Figure S4).

### 3.3. Immunophenotypic Comparison Between CAR-T and Endogenous T Cells at Peak Expansion

**Figure 2:**
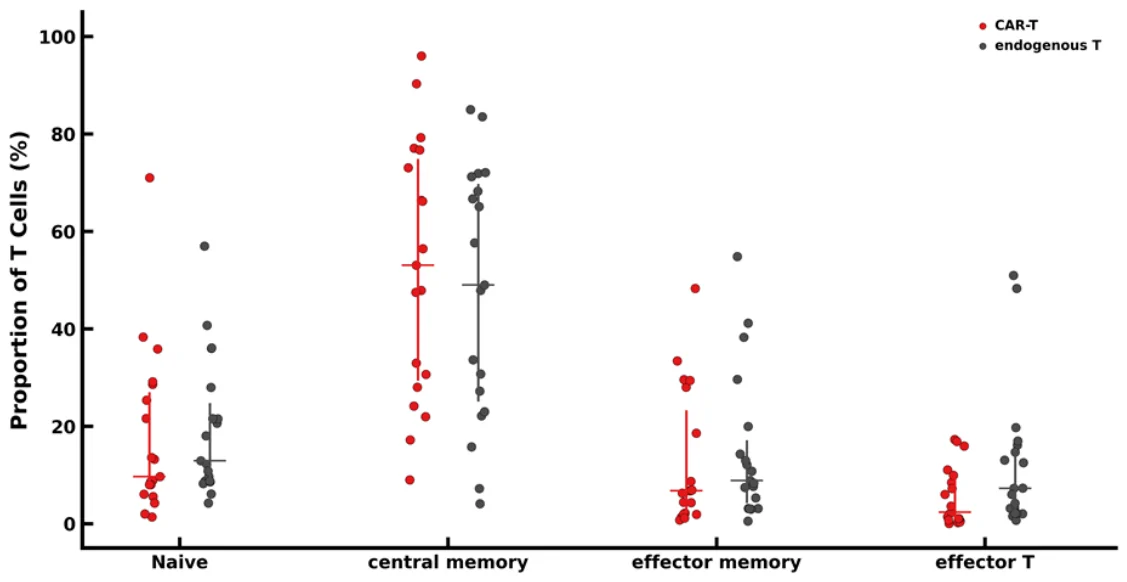
Immunophenotypic comparison between CAR-T cells and endogenous T cells at peak expansion. Comparison of T-cell differentiation subsets between CAR-T cells and endogenous T cells at the individual peak expansion time point. Horizontal bars indicate the median and interquartile range.

**Figure 3:**
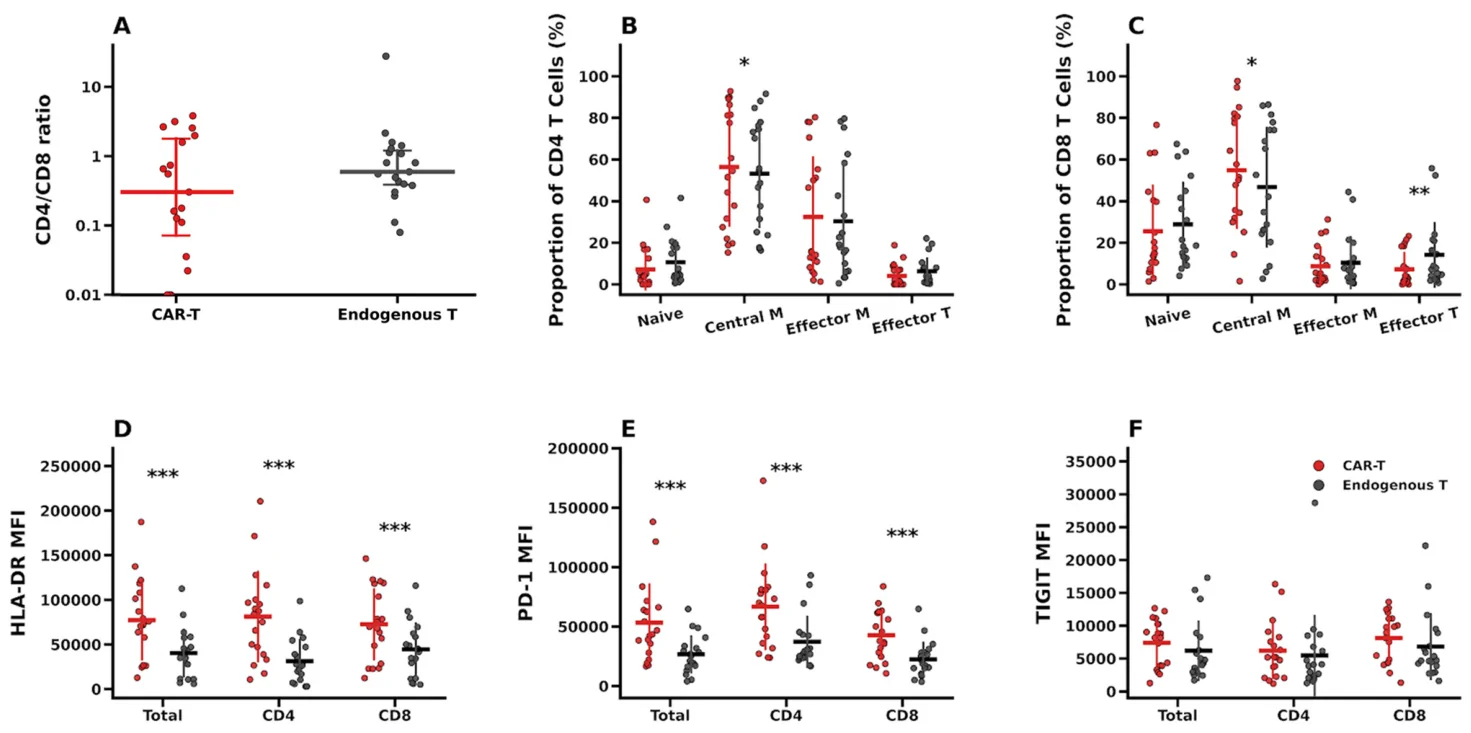
Phenotypic characteristics of CAR-T cells and endogenous T cells at peak expansion. (**A**) CD4/CD8 ratio. (**B**) CD4 T-cell differentiation subsets. (**C**) CD8 T-cell differentiation subsets. (**D**–**F**) Median fluorescence intensity (MFI) of HLA-DR (**D**), PD-1 (**E**), and TIGIT (**F**) on total, CD4+, and CD8+ T cells. Horizontal bars indicate the median. Statistical comparisons were performed using the Wilcoxon signed-rank test. * *p* < 0.05, ** *p* < 0.01, *** *p* < 0.001.

At peak expansion, CAR-T and endogenous T cells exhibited comparable differentiation profiles. TCM cells were the predominant subset in both compartments, (53.05% vs. 48.99%, *p* = 0.726), followed by TN (9.66% vs. 12.89%, *p* = 0.381), TEM (6.74% vs. 8.85%, *p* = 0.255), and TE cells (2.35% vs. 7.23%, *p* = 0.050) (Figure 2). Composite differentiation indices, including memory fraction, effector fraction, and early differentiation index, did not significantly differ between the two compartments (all *p* > 0.05). The CD4/CD8 ratio did not differ significantly between CAR-T and endogenous T cells (median, 0.19 vs. 0.34, *p* = 0.441), with both compartments showing CD8 predominance (Figure 3A). In lineage-specific analyses, CD4+ CAR-T cells contained fewer TN cells than endogenous CD4+ T-cells (median, 3.6% vs. 4.4%, *p* = 0.022), whereas CD8+ CAR-T cells were enriched for TCM cells and depleted of TEM cells compared with endogenous CD8+ T-cells (51.5% vs. 42.1% *p* = 0.018 and 2.8% vs. 7.9% *p* = 0.005, respectively; Figure 3B,C). Expression of HLA-DR, PD-1, and TIGIT was further compared using MFI. HLA-DR and PD-1 expression were significantly higher in CAR-T cells than in endogenous T cells in the total CD3+ compartment (median MFI 72,646 vs. 39,228 and 43,432 vs. 25,358, respectively; both *p* < 0.001), with consistent differences in the CD4+ and CD8+ subsets. TIGIT expression levels did not significantly differ between the two populations in any subset (all *p* > 0.14) (Figure 3D–F).

### 3.4. Changes in CAR-T-Cell Differentiation After Peak Expansion

**Figure 4:**
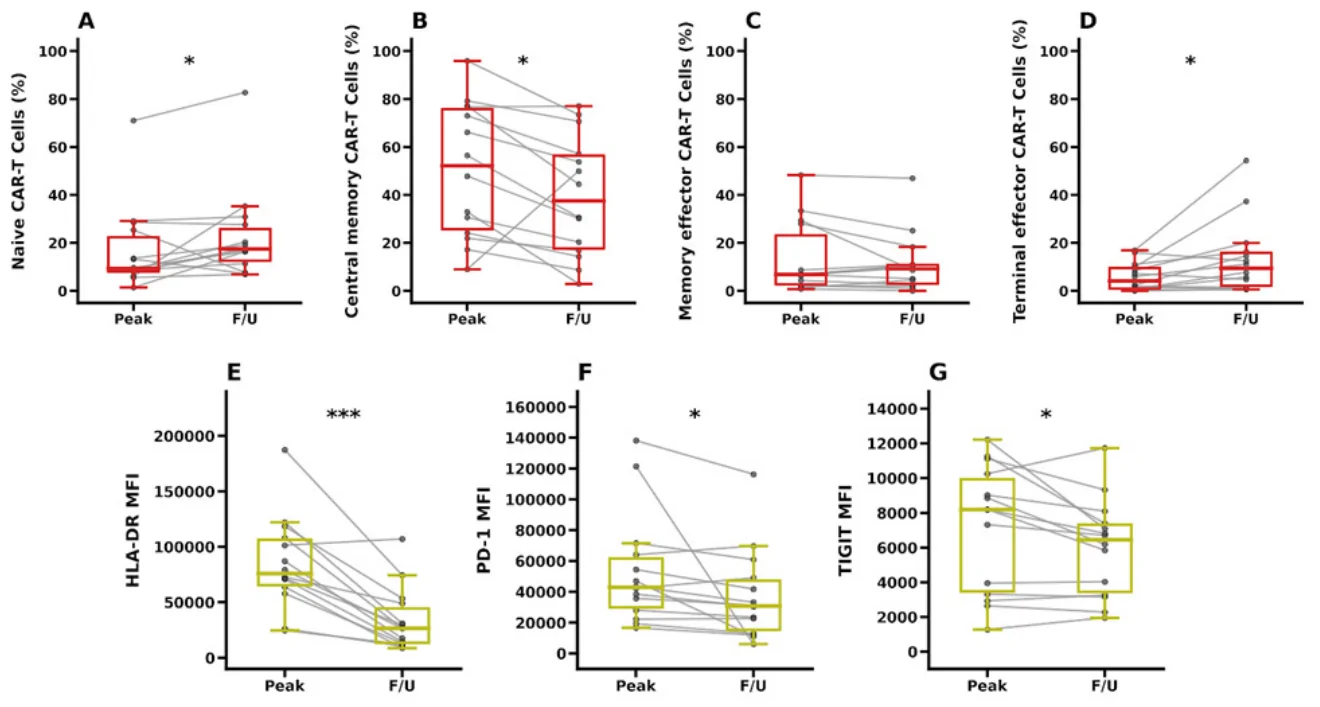
Longitudinal phenotypic remodeling of CAR-T cells following peak expansion. Paired comparison of CAR-T cells at peak expansion (Peak) and the latest available follow-up sample (F/U) (*n* = 14). (**A**–**D**) Frequencies of differentiation subsets. (**E**–**G**) Median fluorescence intensity (MFI) of HLA-DR, PD-1, and TIGIT. Each line represents an individual patient. Box plots indicate the median and interquartile range, with whiskers representing the minimum and maximum values. Statistical comparisons were performed using the Wilcoxon signed-rank test. * *p* < 0.05, , *** *p* < 0.001.

Paired analyses of peak and follow-up samples from 14 patients demonstrated phenotypic changes in CAR-T cells following peak expansion (Figure 4A–D). The TCM cell proportion significantly declined from 52.2% (25.8–75.8) at peak expansion to 37.6% (17.7–56.4) at follow-up (*p* = 0.0166), whereas TE cell proportion increased from 4.2% (0.9–9.6) to 9.4% (2.2–15.9) (*p* = 0.0203). TN cell proportion also modestly increased (9.3% vs. 17.5%, *p* = 0.0419), whereas TEM cell proportions remained unchanged (*p* = 0.715). Consistent with these phenotypic changes, HLA-DR, PD-1, and TIGIT expression significantly declined during follow-up (*p* = 0.0002, *p* = 0.0107, and *p* = 0.0353, respectively, Figure 4E–G).

### 3.5. Exploratory Analysis of T-Cell Phenotypes According to Response and Toxicity

Because all B-ALL patients achieved CR at D30, response-associated analyses were restricted to the DLBCL cohort. Given the limited size of the cohort, toxicity analyses were performed in the pooled cohort including both DLBCL and B-ALL patients. Among patients with DLBCL, responders showed trends toward higher endogenous CD4+ T-cell proportions (31.3% vs. 17.4%, *p* = 0.082) and higher CD8+ TEM cell frequencies (9.0% vs. 2.7%, *p* = 0.082), although none of these differences reached statistical significance. Patients with CRS showed higher CAR-T CD4 TE-cell fractions (4.35% vs. 0%, *p* = 0.081) and increased TIGIT expression on CAR-T cells (41,450 vs. 8854, *p* = 0.094) than those without CRS; however, these differences did not reach statistical significance. In the exploratory ICANS analysis, the two patients with ICANS had higher absolute CAR-T counts than those without ICANS (805 vs. 196 × 10^6^/L, *p* = 0.012). In contrast, lower TIGIT expression was observed in CAR-T CD8+ T cells among patients with ICANS (2685 vs. 9780, *p* = 0.023). Although disease-specific confounding cannot be excluded given the small number of ICANS cases, neither parameter differed significantly between the DLBCL and B-ALL cohorts (absolute CAR-T-cell count, *p* = 0.302; CAR-T CD8+ T-cell TIGIT MFI, *p* = 0.592). Detailed data are shown in Supplementary Table S3.

## 4. Discussion

The present study provides a comprehensive longitudinal characterization of immune remodeling following tisagenlecleucel therapy using multiparameter flow cytometry. By simultaneously monitoring CAR-T cells and endogenous T cells, we demonstrated dynamic immunophenotypic changes associated with CAR-T expansion, contraction, and clinical outcomes. These findings support the utility of integrated flow cytometric immune monitoring for characterizing post-infusion immune remodeling beyond simple CAR-T-cell enumeration.

Most patients exhibited expansion kinetics similar to those reported previously [9,11,19], with CAR-T cells reaching peak expansion predominantly around day 7 after infusion. Delayed peak expansion was observed in several patients with B-ALL, suggesting greater heterogeneity in CAR-T kinetics than in patients with DLBCL. This difference may reflect disease-specific differences in antigen burden and the immune microenvironment, which influence the timing of in vivo CAR-T activation and proliferation.

At peak CAR-T-cell expansion, the CAR-T population showed CD8+ predominance, consistent with previous reports [20,21]. TCM cells represented the predominant differentiation subset in our cohort, in contrast to previous reports describing enrichment of TEM cells or differentiated effector-like phenotypes [5,11,22]. This discrepancy may be attributable to the differences in CAR constructs, disease settings, phenotypic definitions, analytical platforms, and sampling time points.

HLA-DR and PD-1 expression was higher in CAR-T cells than in endogenous T cells at peak expansion, suggesting greater activation-associated phenotypic changes within the CAR-T compartment. Longitudinally, TCM levels declined after peak expansion, accompanied by an increase in TE cells and declining HLA-DR, TIGIT, and PD-1 expression levels. Our observations partially align with those of Odak et al., who reported largely stable activation and checkpoint marker expression over time, with selective decreases in TIM-3, CD95, ICOS, and TIGIT in CD4+ CAR-T-cells during later follow-up [23]. Together, these findings suggest dynamic phenotypic evolution of CAR-T cells from a TCM-predominant, activation-associated state at peak expansion toward a more differentiated phenotype during the post-peak phase [7,24], although their functional implications remain uncertain.

An additional finding of this study was the broadly similar differentiation profile observed between CAR-T cells and endogenous T cells at peak expansion. Although the activation-associated marker expression was more pronounced in the CAR-T compartment, our findings suggest that immune remodeling following CAR-T infusion is not only confined to the CAR-T-cells but also involves coordinated phenotypic changes within endogenous T-cell populations [5]. This observation is consistent with recent single-cell studies demonstrating dynamic interactions between CAR-T cells and the host immune compartment during immune reconstitution [14,15].

Our exploratory analysis did not identify consistent associations between immunophenotypic features and treatment response or treatment-related toxicities. Similarly, Odak et al. reported no significant associations between conventional CAR-T-cell immunophenotypic markers and CRS or ICANS, although exploratory clustering revealed distinct phenotypic patterns in patients with ICANS [23]. Notably, patients who developed ICANS in our cohort exhibited higher absolute CAR-T-cell counts, consistent with previous reports linking greater in vivo CAR-T expansion with an increased risk of neurotoxicity [25,26,27]. However, given the limited number of ICANS cases, the clinical significance of this finding remains uncertain. Together, these observations underscore the challenges of identifying reliable toxicity-associated biomarkers [28,29].

This study has several limitations. First, it was conducted at a single center and included only patients treated with tisagenlecleucel, which may limit the generalizability of the findings. Second, the limited sample size and variable post-peak sampling intervals may have introduced uncertainty and timing-related bias. In addition, differences in baseline characteristics between B-ALL and DLBCL may have confounded the pooled toxicity analyses. These exploratory findings require validation in larger disease-specific cohorts with standardized sampling schedules. Third, analytical validation was limited to cross-reagent comparison and background staining assessment, without systematic evaluation of analytical sensitivity or reproducibility. In addition, viability staining was not performed, potentially allowing interference from nonviable cells. Further assay validation is therefore warranted. Finally, although multiparameter flow cytometry enabled detailed longitudinal immunophenotypic characterization, it could not directly assess the transcriptional or functional states of CAR-T cells and endogenous T cells. Thus, changes in markers such as PD-1, HLA-DR, and TIGIT should be interpreted as phenotypic observations rather than direct evidence of functional activation or exhaustion [30,31].

Nevertheless, this study provides a comprehensive longitudinal flow cytometric analysis to simultaneously evaluate both CAR-T and endogenous T-cell compartments in a real-world Korean cohort receiving commercial tisagenlecleucel therapy.

## 5. Conclusions

Our findings demonstrate that multiparameter flow cytometry can capture dynamic immune remodeling beyond simple CAR-T enumeration and support its potential role as a practical platform for longitudinal immune monitoring following CAR-T therapy.

## Figures and Tables

**Table 1:** Baseline characteristics, early clinical outcomes, and CAR-T-cell collection characteristics of the study cohort.

Variable	Total Cohort	B-ALL (*n* = 8)	DLBCL (*n* = 11)	*p* Value
**Baseline characteristics at CAR-T infusion**				
Age	41.00 (13.00–59.00)	13.00 (11.50–14.00)	59.00 (54.50–67.50)	**<0.001**
Sex (Male)	13 (68.4%)	4 (50.0%)	9 (81.8%)	0.319
WBC (10^9^/L)	1.69 (0.60–5.49)	0.35 (0.08–1.88)	3.01 (1.55–7.27)	**0.012**
RBC (10^12^/L)	3.08 (2.86–3.60)	2.88 (2.70–3.03)	3.29 (3.00–3.75)	0.051
Hb (g/dL)	9.40 (8.40–10.80)	8.55 (7.78–9.00)	10.60 (9.55–11.35)	**0.023**
Hct (%)	29.30 (24.80–32.80)	24.75 (23.52–27.00)	32.50 (29.30–33.55)	**0.017**
Platelet count (10^9^/L)	111.00 (38.50–150.50)	91.50 (21.75–153.25)	111.00 (89.00–143.50)	0.563
Neutrophil (%)	92.00 (80.00–95.30)	85.00 (47.50–93.50)	93.70 (87.00–95.30)	0.282
Lymphocytes (%)	2.00 (0.70–9.35)	15.00 (3.25–43.50)	1.70 (0.35–2.50)	**0.032**
Monocytes (%)	2.00 (0.55–5.00)	0.20 (0.00–4.25)	2.70 (1.80–5.00)	0.146
ANC (10^9^/L)	1.50 (0.58–4.97)	0.31 (0.04–1.19)	2.82 (1.35–6.78)	**0.009**
ALC (10^9^/L)	0.05 (0.02–0.07)	0.03 (0.01–0.04)	0.06 (0.04–0.07)	0.105
LDH (U/L)	242 (160–294)	160 (146.75–230.50)	264 (236–475.50)	**0.016**
NGS-MRD (%)	-	46.92 (19.41–81.63)	-	-
**Early clinical outcomes**				
CRS grade ≥ 2	10 (52.6%)	3 (37.5%)	7 (63.6%)	0.370
ICANS presence	2 (10.5%)	0 (0.0%)	2 (18.2%)	0.485
D30 response	14 (73.7%)	8 (100.0%)	6 (54.5%)	0.045
CMR	4 (21.1%)	0 (0.0%)	4 (36.4%)	
PMR	2 (10.5%)	0 (0.0%)	2 (18.2%)	
PD	5 (26.3%)	0 (0.0%)	5 (45.5%)	
CR	8 (42.1%)	8 (100.0%)	0 (0.0%)	
**CAR-T collection characteristics**				
TNC (10^9^)	23.77 (12.18–34.36)	9.14 (6.73–51.31)	26.66 (22.20–34.36)	0.177
CD3+ (10^9^)	8.83 (5.22–14.02)	5.22 (3.52–8.53)	10.41 (8.76–15.77)	**0.029**
CD3+/TNC ratio (%)	47.96 (30.52–55.84)	44.77 (18.11–78.58)	47.96 (41.45–53.03)	0.904
Apheresis volume (mL)	270.00 (267.50–277.00)	270.00 (263.00–274.75)	271.00 (268.00–277.00)	0.59

Data are presented as median (interquartile range) or *n* (%). Continuous variables were compared using the Mann–Whitney U test and categorical variables using Fisher’s exact test. Bold values indicate statistical significance (*p* < 0.05). DLBCL response was assessed using the Lugano classification, whereas B-ALL response was assessed according to standard hematologic response criteria. Abbreviations: DLBCL, diffuse large B-cell lymphoma; B-ALL, B-cell acute lymphoblastic leukemia; n, number of patients; WBC, white blood cell; RBC, red blood cell; Hb, hemoglobin; Hct, hematocrit; ANC, absolute neutrophil count; ALC, absolute lymphocyte count; LDH, lactate dehydrogenase; NGS-MRD, next-generation sequencing-based measurable residual disease using LymphoTrack assay panels (InVivoScribe, San Diego, CA, USA); CRS, cytokine release syndrome; ICANS, immune effector cell-associated neurotoxicity syndrome; CMR, complete metabolic response; PMR, partial metabolic response; PD, progressive disease; CR, complete remission; TNC, total nucleated cell.

## Data Availability

Flow cytometry data and the raw FSC files are available on reasonable request from the corresponding author (J.M.L.).

## Supplementary Materials

The following supporting information can be downloaded at: https://www.mdpi.com/article/10.3390/cancers18203374/s1. Figure S1. Comparison of two CAR-T detection reagents; Figure S2. Assessment of background staining in CAR-T-cell-negative samples; Figure S3. Flow cytometric gating strategy for identification and immunophenotypic characterization of CAR-T cells and endogenous T cells; Figure S4. Longitudinal CAR-T-cell expansion kinetics following infusion; Table S1. Antibodies used for flow cytometric detection and immunophenotypic characterization of CD19 CAR-T cells; Table S2. Patient-level time points included in the paired longitudinal analysis; Table S3. Exploratory analysis of CAR-T and endogenous T-cell phenotypes according to clinical response and treatment-related toxicities.

## Abbreviations

The following abbreviations are used in this manuscript:

CAR	Chimeric Antigen Receptor
B-ALL	B-cell Precursor Acute Lymphoblastic Leukemia
DLBCL	Diffuse Large B-cell Lymphoma
CRS	Cytokine Release Syndrome
ICANS	Immune Effector Cell-associated Neurotoxicity Syndrome
CTIWP	Cellular Therapy and Immunobiology Working Party
EBMT	European Society for Blood and Marrow Transplantation
PBS	Phosphate-Buffered Saline
FSC-H	Forward Scatter Height
FSC-A	Forward Scatter Area
SSC-A	Side Scatter Area
TN	Naïve T-cells
TCM	Central Memory T-cells
TEM	Effector Memory T-cells
TE	Effector T-cells
MFI	Median Fluorescence Intensity
CR	Complete Remission
CMR	Complete Metabolic Response
PMR	Partial Metabolic Response
PD	Progressive Disease
