# Supplementary material for: Longitudinal Flow Cytometric Profiling of CAR-T and Endogenous T Cells Following CD19 CAR-T Therapy
Source: Cancers (Basel). 2026 Oct 19;18(20):3374. doi: 10.3390/cancers18203374 (PMC13702454; doi:10.3390/cancers18203374)
Supplement: Supplementary file 1 [file cancers-18-03374-s001.zip › cancers-4635103-supplementary.pdf]

Supplementary Figure S1. Comparison of two CAR-T detection reagents.

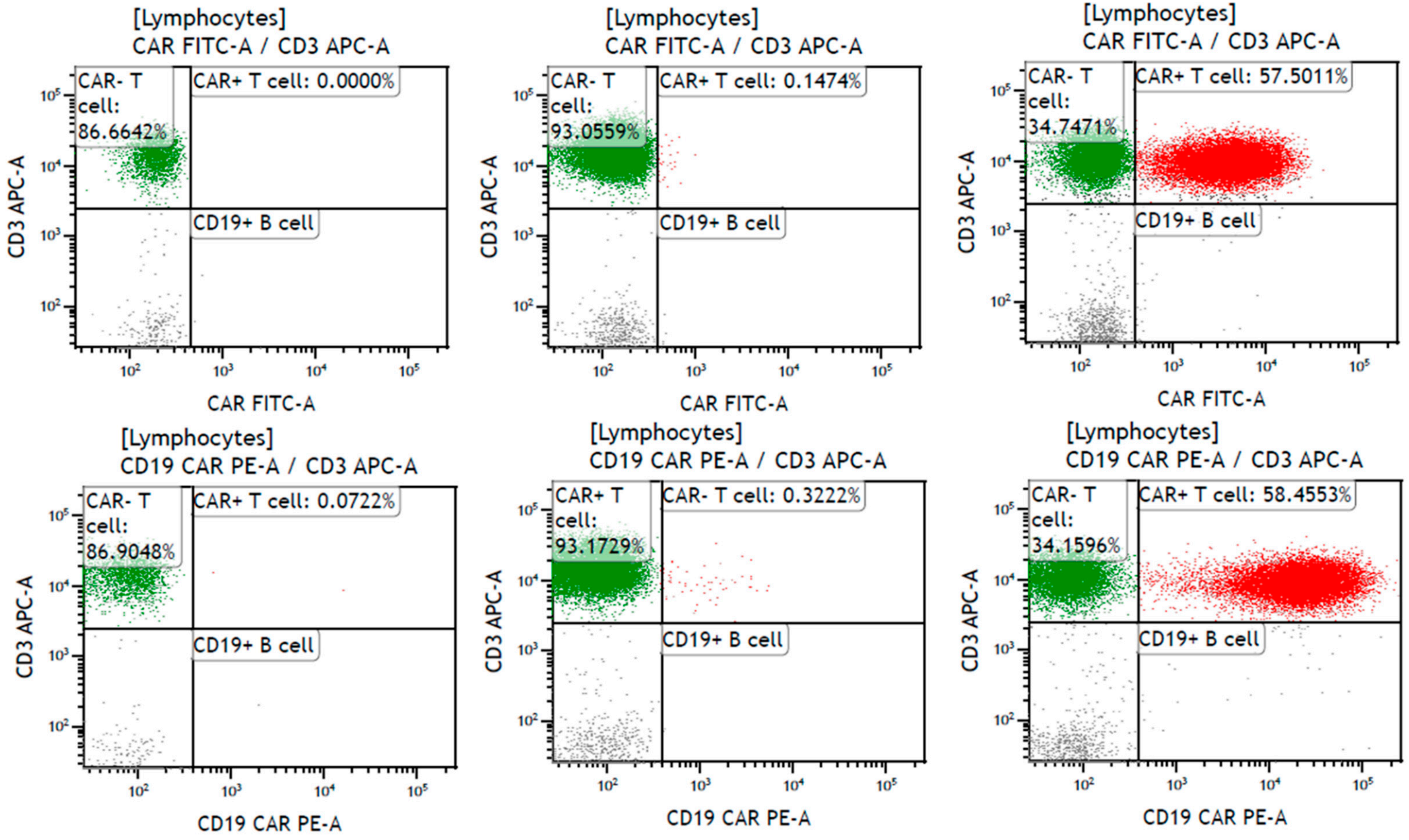

Representative flow cytometry plots demonstrating CAR-T cell detection using two independent anti-CAR reagents (CAR-FITC using FITC-Labeled Monoclonal Anti-FMC63 Antibody [Mouse IgG1, clone Y45, premium grade; Acrobiosystems], upper panels; CD19 CAR-PE using PE CD19 CAR Detection Reagent [BD Biosciences], lower panels). Three representative samples with absent (left), low (middle), and high (right) circulating CAR-T cell frequencies are shown. Both reagents identified comparable CAR-positive T-cell populations across the full range of CAR-T cell frequencies, supporting the analytical concordance of the staining strategy.

Supplementary Figure S2. Assessment of background staining in CAR-T-cell-negative samples.

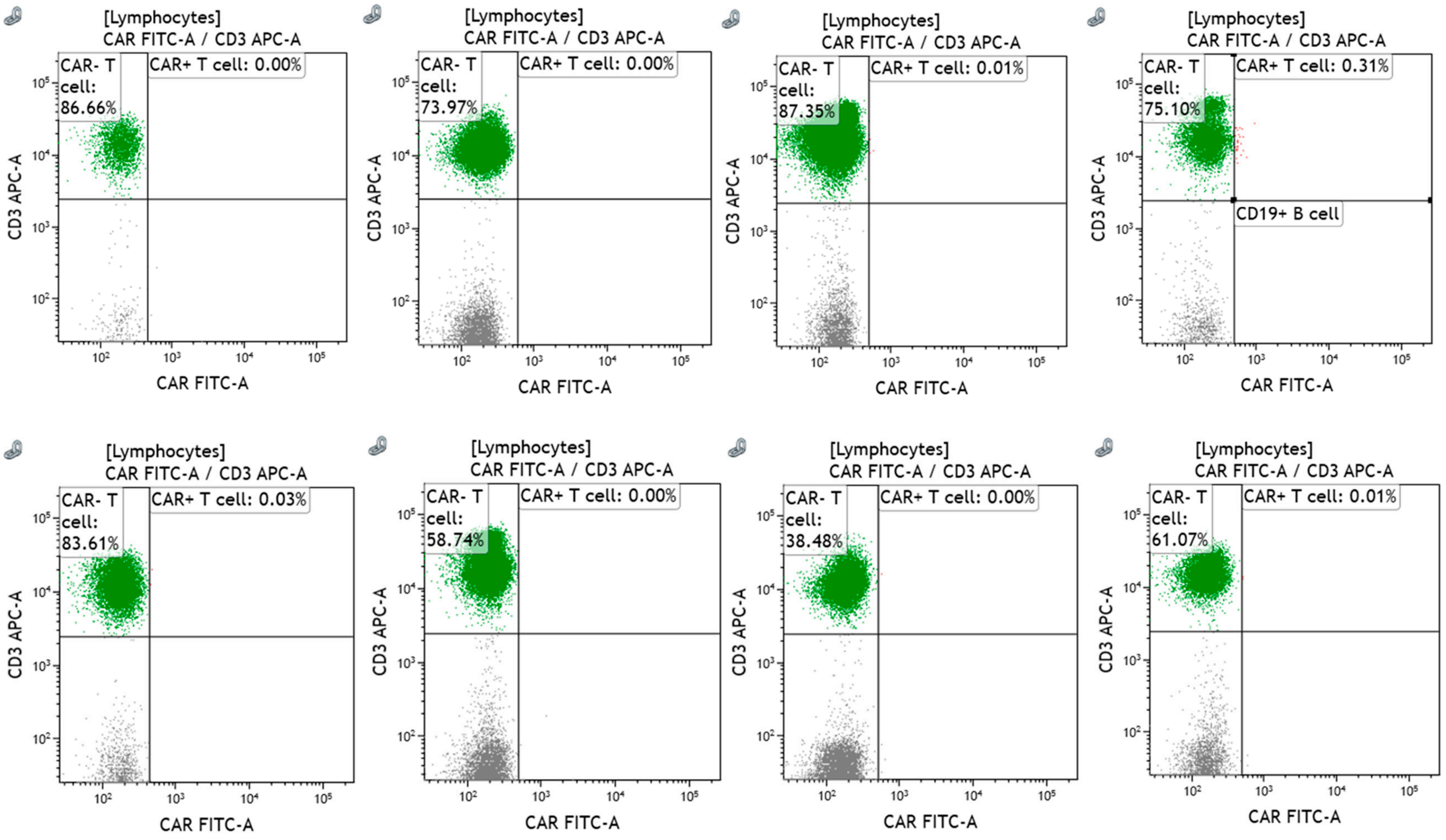

Representative flow cytometry plots from eight CAR-T-cell-negative samples, including one previously evaluated negative control and seven additional samples, analyzed using the study CAR detection reagent. CAR-positive T-cell proportions were expressed as percentages of total $CD3^{+}$ T cells. Background CAR-positive staining was minimal (median, 0.005%; range, 0–0.31%). The gating thresholds established using these negative controls were consistently applied to all study samples.

Supplementary Figure S3. Flow cytometric gating strategy for identification and immunophenotypic characterization of CAR-T cells and endogenous T cells.

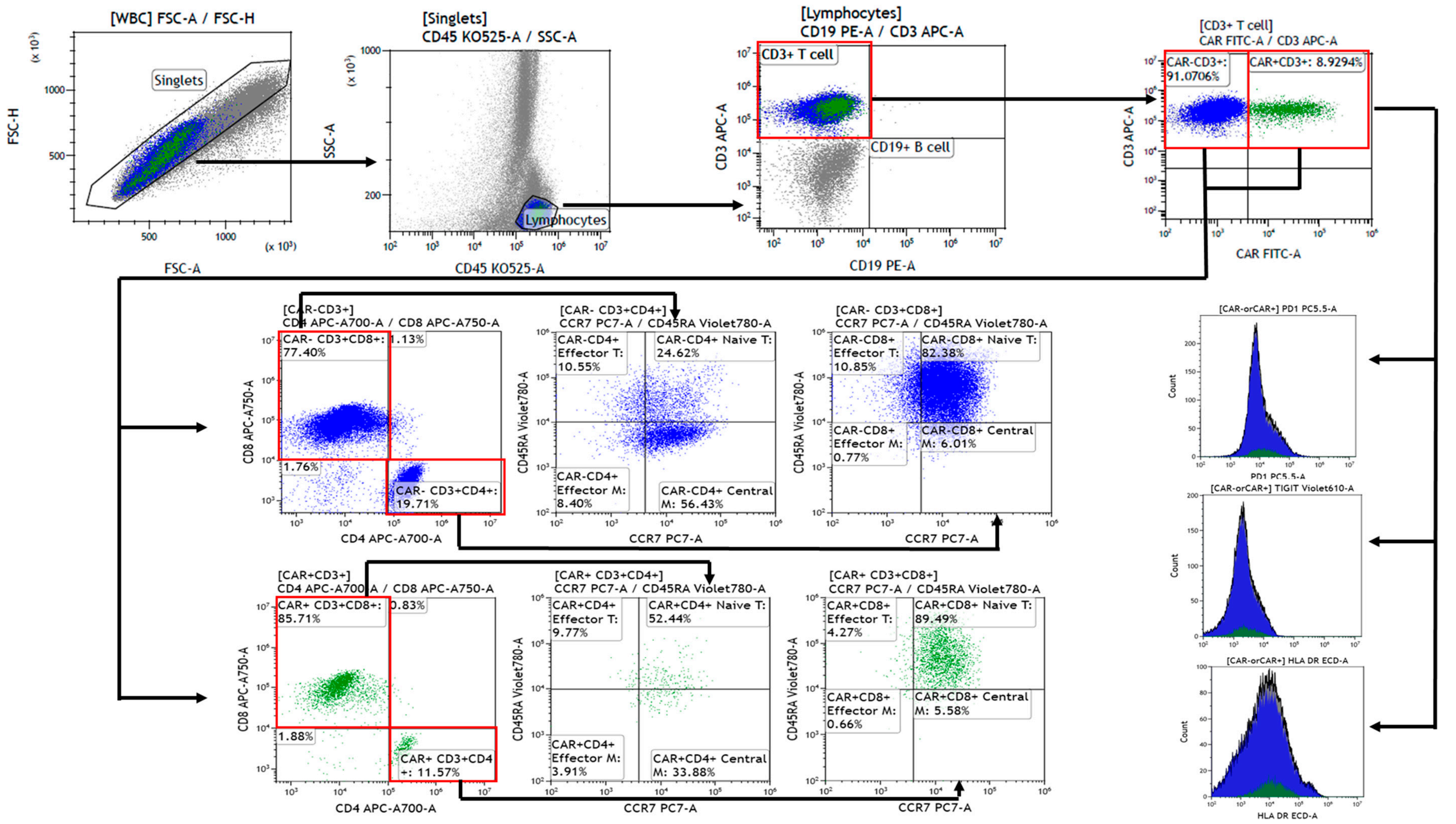

Representative gating strategy used for flow cytometric analysis. After exclusion of doublets, lymphocytes were identified based on CD45 expression and side scatter characteristics. CD3-positive T cells were subsequently separated into CAR-positive (CAR+) and CAR-negative (CAR−) populations. Both CAR+ and CAR− T cells were further subdivided into CD4+ and CD8+ subsets. T-cell differentiation subsets were defined according to CD45RA and CCR7 expression as naïve (TN), central memory (TCM), effector memory (TEM), and terminal effector (TE) T cells. Expression of HLA-DR, PD-1, and TIGIT was evaluated in each T-cell subset.

Supplementary Figure S4. Longitudinal CAR-T-cell expansion kinetics following infusion.

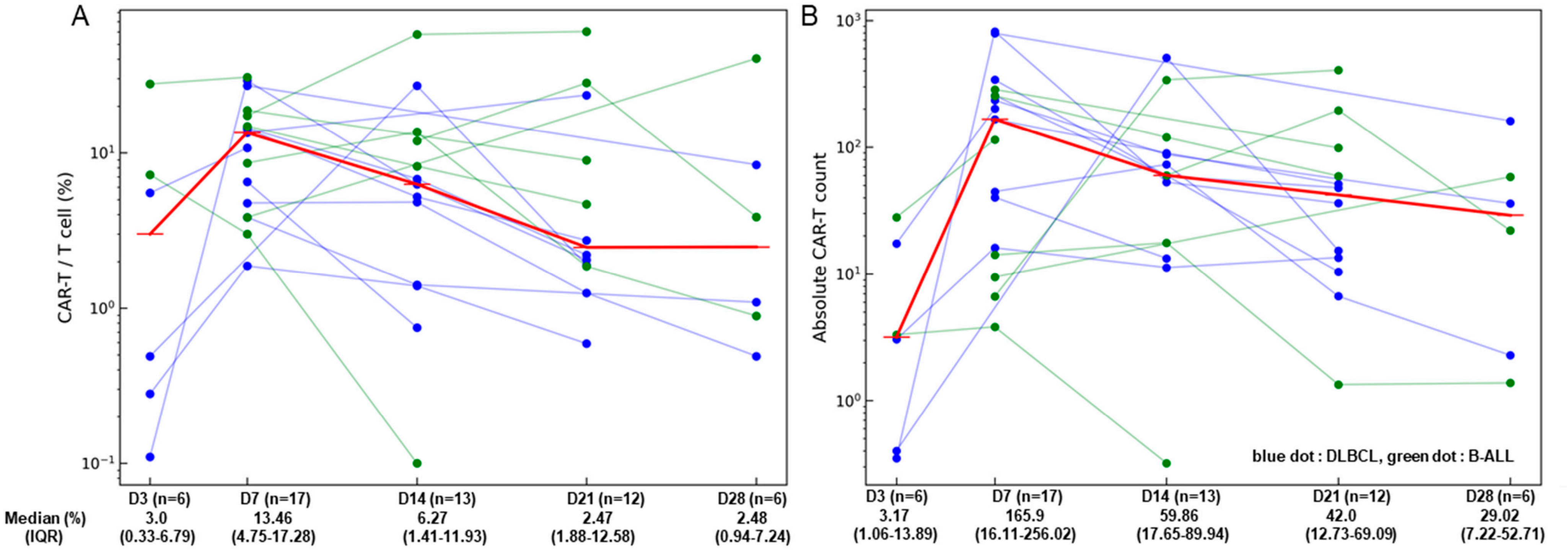

(A) Relative CAR-T-cell proportion among total CD3+ T cells (%). (B) Absolute circulating CAR-T-cell counts ($\times 10^{6}$/L) during the first 28 days after infusion. Each line represents an individual patient; blue indicates patients with DLBCL, green indicates patients with B-ALL, and red indicates the median values.

Supplementary Table S1. Antibodies used for flow cytometric detection and immunophenotypic characterization of CD19 CAR-T cells.

Antibody	Fluorochrome	Company	Reference
CD45	KrO	Beckman Coulter	A96416
TIGIT	Violet610	Biolegend	372712
CD45RA	Violet780	Biolegend	304140
CAR Detection	FITC	Acrobiosystems	FM3-FY45G0
CD19	PE	Beckman Coulter	IM1285U
HLA DR	ECD	Beckman Coulter	IM3636
PD1 (CD279)	PC5.5	Beckman Coulter	B36123
CCR7 (CD197)	PC7	Beckman Coulter	B46025
CD3	APC	Beckman Coulter	IM2467U
CD4	APC-A700	Beckman Coulter	B10824
CD8	APC-A750	Beckman Coulter	A94686

Abbreviations: CAR, chimeric antigen receptor; FITC, fluorescein isothiocyanate; PE, phycoerythrin; ECD, phycoerythrin-Texas Red; APC, allophycocyanin; APC-A700, allophycocyanin-Alexa Fluor 700; APC-A750, allophycocyanin-Alexa Fluor 750; KrO, Krome Orange; PC5.5, phycoerythrin-cyanine 5.5; PC7, phycoerythrin-cyanine 7.

Supplementary Table S2. Patient-level time points included in the paired longitudinal analysis.

Patient ID	Diagnosis	Peak day	CAR-T at peak (%)	Follow-up day	CAR-T at follow-up (%)	Peak-to-follow-up interval (days)
1	DLBCL	D7	14.26	D21	2.73	14
2	DLBCL	D7	3.85	D28	1.09	21
3	DLBCL	D7	1.87	D21	0.59	14
4	DLBCL	D14	27.05	D21	1.89	7
6	DLBCL	D7	6.49	D14	0.75	7
7	DLBCL	D7	14.46	D21	2.20	14
8	DLBCL	D7	27.06	D28	8.37	21
9	DLBCL	D7	29.22	D21	2.05	14
11	DLBCL	D14	4.81	D28	0.49	14
12	B-ALL	D3	7.22	D14	0.10	11
13	B-ALL	D7	18.78	D21	8.93	14
16	B-ALL	D14	13.62	D28	0.89	14
18	B-ALL	D21	28.14	D28	3.87	7
19	B-ALL	D7	14.82	D21	4.67	14

Fourteen patients with at least one available sample after their individual peak CAR-T expansion were included. Peak and follow-up samples correspond to the paired time points used for longitudinal phenotypic comparisons. Because sample availability varied among patients, the peak and follow-up time points were not uniform across the cohort. Missing scheduled samples were not imputed. DLBCL, diffuse large B-cell lymphoma; B-ALL, B-cell acute lymphoblastic leukemia.

Supplementary Table S3. Exploratory analysis of CAR-T and endogenous T-cell phenotypes according to clinical response and treatment-related toxicities.

T cell subset			Responder	Non-responder	P-value	CRS grade ≥2	CRS grade <2	P-value	ICANS presence	ICANS absence	P-value	DLBCL	B-ALL	P-value
CAR-T	relative		13.90 (8.25-23.90)	10.70 (3.90-14.50)	0.46	22.95 (14.35-27.85)	13.50 (6.50-14.80)	0.31	28.15 (27.62-28.68)	14.30 (6.50-27.10)	0.23	13.50 (5.65–20.80)	23.45 (14.50–33.15)	0.08
	absolute		298.20 (118.68-679.32)	201.30 (166.00-234.90)	0.43	270.90 (173.38-484.12)	114.80 (40.20-234.90)	0.08	805.25 (798.77-811.73)	195.50 (58.30-256.00)	0.01	235 (119–425)	155 (48–262)	0.30
	CD4		35.35 (26.15-44.77)	22.00 (6.20-52.00)	0.79	34.05 (4.58-48.72)	14.40 (7.60-22.00)	0.6	34.05 (29.77-38.33)	14.40 (6.20-45.50)	0.49	28.10 (18.35–47.65)	8.65 (2.20–11.47)	0.06
	CD8		61.25 (49.10-69.28)	68.20 (43.70-79.30)	1	50.60 (44.25-82.67)	79.30 (68.90-83.40)	0.39	61.50 (57.55-65.45)	76.50 (45.90-86.70)	0.75	68.20 (45.65–74.35)	84.75 (68.85–87.62)	0.20
	CD4/CD8		0.16 (0.05–0.70)	0.32 (0.08–1.19)	0.82	0.58 (0.05–1.13)	0.11 (0.08–0.32)	0.44	0.58 (0.48–0.69)	0.12 (0.03–0.96)	0.46	0.40 (0.20–1.12)	0.10 (0.02–0.14)	0.17
	CD4	TN	3.60 (0.90-4.12)	1.20 (0.80-2.00)	0.78	3.60 (1.10-10.45)	4.30 (0.00-5.90)	1	3.95 (3.77-4.12)	3.60 (0.00-12.50)	0.95	2.00 (0.40–3.95)	14.65 (4.43–17.42)	0.04
		TCM	81.35 (44.00-87.20)	51.50 (37.80-86.30)	0.93	41.00 (28.52-76.08)	77.60 (51.50-89.10)	0.35	56.40 (44.00-68.80)	54.50 (27.50-86.30)	0.95	81.20 (34.70–87.70)	49.35 (21.12–64.92)	0.30
		TE	0.20 (0.00-1.30)	0.80 (0.40-2.00)	0.51	4.35 (0.70-8.43)	0.00 (0.00-1.40)	0.08	5.55 (3.58-7.53)	0.80 (0.00-6.80)	0.31	0.40 (0.00–1.80)	6.75 (1.05–8.50)	0.19
		TEM	13.50 (11.40-45.00)	46.50 (12.50-51.20)	1	32.90 (12.60-54.27)	14.10 (5.10-46.50)	0.35	34.10 (23.50-44.70)	15.60 (8.20-51.20)	0.75	14.10 (11.70–53.25)	15.70 (6.65–57.00)	0.84
	CD8	TN	14.40 (11.30-19.00)	10.50 (5.80-13.30)	0.46	18.85 (13.40-40.23)	14.80 (7.30-24.90)	0.46	27.05 (20.38-33.73)	15.10 (10.50-39.70)	0.74	13.30 (8.15–17.70)	32.30 (16.75–49.20)	0.05
		TCM	70.90 (54.67-79.10)	82.10 (80.70-85.10)	0.33	54.60 (35.85-75.75)	50.20 (34.40-80.70)	1	57.85 (54.67-61.03)	50.20 (31.90-80.70)	0.84	79.60 (57.85–83.60)	35.05 (26.02–48.33)	0.01
		TE	2.25 (1.02-3.40)	1.30 (0.00-2.40)	0.41	3.20 (0.85-8.38)	2.40 (1.70-18.30)	0.84	3.20 (3.00-3.40)	2.40 (1.00-18.30)	0.95	1.70 (0.65–3.20)	12.85 (3.70–18.77)	0.05
		TEM	7.40 (5.30-16.10)	3.20 (2.00-4.60)	0.18	4.90 (2.08-12.35)	2.70 (1.90-12.20)	0.9	11.80 (8.50-15.10)	3.20 (1.70-12.20)	0.42	5.20 (2.60–10.70)	2.20 (1.43–16.38)	0.59

	CAR-T	PD1	38961.00 (29952.25-43128.50)	54352.00 (17539.00-64010.00)	0.93	39568.00 (23640.75-63644.75)	46924.00 (39076.00-66196.00)	0.44	28940.00 (25558.00-32322.00)	46924.00 (38414.00-66196.00)	0.23	42,218 (25,106–59,181)	54,736 (38,910–93,164)	0.20
		TGIT	6375.50 (3465.00-8967.25)	7318.00 (3895.00-11122.00)	0.66	4145.00 (3017.25-9519.25)	8854.00 (8193.40-10222.00)	0.09	2603.50 (1934.25-3272.75)	8809.00 (4348.00-10253.00)	0.11	7,318 (3,600–9,636)	8,524 (7,221–10,481)	0.43
		HLA-DR	86982.50 (71848.75-116876.25)	86979.00 (79232.00-108118.00)	0.79	72114.50 (35536.00-85042.25)	75857.00 (57888.00-118414.00)	0.49	97354.00 (85000.00-109708.00)	71583.00 (26702.00-101319.00)	0.42	86,979 (74,252–113,266)	42,295 (24,576–65,507)	0.02
	CD4	PD1	64081.00 (38406.00-72588.50)	77756.00 (24081.00-83370.00)	0.79	53019.00 (32903.50-69571.50)	80786.00 (67790.00-83370.00)	0.11	50285.50 (38721.75-61849.25)	67790.00 (41709.00-81242.00)	0.57	70,115 (29,508–79,499)	62,886 (46,469–84,336)	0.65
		TGIT	4452.50 (2467.00-5220.00)	4752.00 (2250.00-5187.00)	1	3773.50 (2052.25-6973.20)	6308.30 (5187.00-8262.00)	0.16	2446.00 (1810.00-3082.00)	5231.00 (3829.00-8262.00)	0.1	2,678 (2,070–4,418)	5,104 (3,431–7,212)	0.39
		HLA-DR	85762.00 (77429.25-109174.25)	95149.00 (89994.00-96926.00)	0.79	79660.50 (37285.00-96481.75)	87401.00 (47094.00-127807.00)	0.6	95815.00 (85506.50-106123.50)	84123.00 (38857.00-96926.00)	0.57	36,162 (26,515–50,864)	8,632 (5,080–19,517)	0.01
	CD8	PD1	32616.00 (25618.25-37697.50)	50154.00 (15535.00-63410.00)	0.79	32616.00 (20227.75-62264.50)	45775.00 (35583.00-56122.00)	0.6	23510.00 (21119.00-25901.00)	45775.00 (32205.00-61542.00)	0.23	36,940 (21,728–55,848)	50,948 (34,738–61,672)	0.39
		TGIT	6489.85 (4400.75-9263.92)	11092.00 (4853.00-12014.00)	0.25	4915.00 (4119.00-10198.42)	9846.00 (8959.00-11061.00)	0.08	2684.50 (2003.25-3365.75)	9780.00 (5462.00-11092.00)	0.02	4,715 (4,550–7,882)	4,618 (2,879–8,436)	0.59
		HLA-DR	86733.00 (65148.50-116572.75)	78603.00 (78526.00-118197.00)	0.79	66691.00 (29680.00-78583.75)	75377.00 (56850.00-118197.00)	0.44	95321.50 (82548.75-108094.25)	68019.00 (28373.00-103690.00)	0.42	50,115 (43,264–74,919)	11,610 (6,516–37,242)	0.01
**Endogeneous T cell**	relative		86.10 (76.10-91.75)	89.30 (85.50-96.10)	0.46	77.05 (72.15-85.65)	86.50 (85.20-93.50)	0.31	71.85 (71.33-72.38)	85.70 (72.90-93.50)	0.23	86.50 (79.20–94.35)	76.55 (66.85–85.50)	0.08
	CD4		31.25 (24.38-36.02)	17.40 (14.80-17.80)	0.08	25.95 (17.57-36.02)	23.30 (17.80-29.90)	0.97	41.45 (38.17-44.73)	23.30 (17.40-29.90)	0.14	23.30 (17.60–35.65)	26.35 (15.33–31.88)	1
	CD8		62.30 (61.62-70.85)	72.30 (69.80-78.40)	0.33	66.75 (61.62-77.77)	69.80 (57.00-75.30)	0.9	54.15 (50.38-57.93)	70.60 (61.60-78.40)	0.23	69.80 (61.65–74.40)	69.10 (52.62–82.35)	0.97
	CD4/CD8		0.44 (0.30-0.64)	0.24 (0.19-0.26)	0.25	0.39 (0.23-0.58)	0.32 (0.26-0.66)	0.93	0.80 (0.68-0.92)	0.32 (0.23-0.59)	0.16	0.31 (0.25–0.57)	0.39 (0.19–0.68)	1
	CD4	TN	4.35 (1.90-4.40)	3.40 (2.50-5.10)	0.93	4.75 (4.33-16.98)	4.30 (2.10-19.40)	0.68	4.40 (4.40-4.40)	5.10 (2.50-17.80)	0.95	4.30 (1.90–4.75)	17.75 (6.77–21.45)	0.04
		TCM	72.35 (36.38-75.78)	54.60 (46.60-78.00)	0.93	42.10 (26.68-69.33)	70.20 (54.60-74.50)	0.35	50.65 (37.88-63.43)	54.60 (31.40-74.50)	1	70.20 (35.85–77.10)	43.15 (29.45–60.08)	0.54

		TE	3.55 (1.52-6.47)	1.50 (1.10-1.70)	0.79	7.65 (1.83-14.78)	2.30 (1.50-4.00)	0.19	6.00 (5.00-7.00)	3.10 (1.10-8.10)	0.6	1.70 (1.05–5.65)	7.90 (3.58–12.12)	0.08
		TEM	20.00 (11.32-53.12)	19.10 (17.80-42.60)	0.79	20.85 (15.57-55.20)	17.80 (3.30-42.60)	0.39	38.95 (27.12-50.78)	19.10 (6.40-42.60)	0.74	19.10 (12.65–52.60)	19.50 (3.30–39.33)	0.54
	CD8	TN	17.50 (15.35-20.70)	11.90 (9.10-12.60)	0.13	18.40 (13.73-44.08)	21.40 (11.90-36.20)	0.65	28.30 (21.65-34.95)	18.60 (12.60-44.90)	0.95	15.00 (12.25–20.00)	40.55 (27.72–54.53)	0.08
		TCM	67.00 (36.03-72.85)	81.60 (77.90-85.90)	0.12	30.50 (21.30-67.90)	52.60 (28.80-74.20)	0.54	44.75 (34.53-54.98)	42.10 (26.20-74.20)	0.74	74.20 (45.75–79.75)	27.50 (8.02–36.55)	0.01
		TE	5.95 (3.20-13.80)	2.40 (1.80-4.10)	0.18	15.25 (5.33-19.77)	5.20 (2.70-8.90)	0.49	15.55 (11.38-19.72)	7.90 (2.70-16.40)	0.49	4.10 (2.15–10.85)	18.65 (8.65–29.23)	0.01
		TEM	9.00 (7.05-12.00)	2.70 (1.40-6.10)	0.08	5.90 (2.75-12.00)	6.10 (2.40-8.50)	0.74	11.40 (10.80-12.00)	4.00 (2.40-8.50)	0.26	6.80 (2.55–9.35)	3.60 (2.40–20.35)	0.93

Data are presented as median (interquartile range). Response analyses were performed only in patients with DLBCL because all patients with B-ALL achieved complete remission at day 30. Toxicity analyses included the entire cohort. P values were calculated using the Mann–Whitney U-test. Abbreviations: CAR-T, chimeric antigen receptor T cell; CRS, cytokine release syndrome; ICANS, immune effector cell-associated neurotoxicity syndrome; DLBCL, diffuse large B-cell lymphoma; B-ALL, B-cell acute lymphoblastic leukemia; TN, naïve T cell; TCM, central memory T cell; TEM, effector memory T cell; TE, terminal effector T cell; PD-1, programmed cell death protein 1; TIGIT, T-cell immunoreceptor with Ig and ITIM domains; HLA-DR, human leukocyte antigen-DR; MFI, median fluorescence intensity.
